# Supplementary material for: National Early Warning Score 2 (NEWS2) to predict poor outcome in hospitalised COVID-19 patients in India
Source: PLoS One. 2021 Dec 15;16(12):e0261376. doi: 10.1371/journal.pone.0261376 (PMC8673675; doi:10.1371/journal.pone.0261376)
Supplement: S1 Table — (DOCX) [file pone.0261376.s001.docx]

**Tables**

**Table 1. 2 by 2 table of sensitivity and specificity of NEWS2 score of 5 or more in predicting poor outcomes**

|  | Good outcome | Poor outcome | Total |
| --- | --- | --- | --- |
| Score less than 5 | 261 | 2 | 263 |
| Score of 5 or more | 108 | 28 | 136 |
| Total | 369 | 30 | 399 |

Sensitivity = 28/30 = 93.3%

Specificity = 261/369 = 70.7%

Positive predictive value = 28/136 = 20.6%

Negative predictive value = 261/263 = 99.2%

**Table 2. 2 by 2 table of sensitivity and specificity of NEWS2 score of 6 or more in predicting poor outcomes**

|  | Good outcome | Poor outcome | Total |
| --- | --- | --- | --- |
| Score less than 6 | 281 | 3 | 284 |
| Score of 6 or more | 88 | 27 | 115 |
| Total | 369 | 30 | 399 |

Sensitivity = 27/30 = 90.0%

Specificity = 305/369 = 76.2%

Positive predictive value = 27/115 = 23.5%

Negative predictive value = 281/284 = 98.9%
