# Supplementary material for: National Early Warning Score 2 (NEWS2) to predict poor outcome in hospitalised COVID-19 patients in India
Source: PLoS One. 2021 Dec 15;16(12):e0261376. doi: 10.1371/journal.pone.0261376 (PMC8673675; doi:10.1371/journal.pone.0261376)
Supplement: Attachment — Submitted filename: Response to Reviewers.docx [file pone.0261376.s003.docx]

**Reviewer 1**

| **Reviewer’s comments** | **Author’s responses** |
| --- | --- |
| 1. One of the major claim by the study is that a NEWS2 score of 5 or more can predict the poor outcome in the case of COVID19 patients better than the NEWS2 score of 6. There is no comparison between score 5 and 6 mentioned in the result section of this study. If the authors claiming so, need to give a comparison between both the scores showing how one is better than other. “The baseline NEWS2 score was 5 or more in 136 of the 399 (34.1%) patients while it was 6 or more in 115 of the 399 (28.8%) patients.” How many poor outcomes were there in 21 extra patients that was identified by score 5? Can this score be used by the doctors for taking any decision? | We thank the reviewer for this observation. We have now included the comparison of diagnostic accuracy measures of the two cut-offs (5 and 6). This has been added as a table in the manuscript.  The sensitivity of score 5 or more was 93.3% compared to 90% for score of 6 or more. Since the score is proposed to be used as a screening tool for identifying patients who are likely to have poor outcomes, we chose the score cut off with higher sensitivity.  This cut off however, identified one poor outcome (death) among the 21 patients.  We believe that when used in the real life setting where case numbers are very high, this might still be relevant. |
| 2. The result section is poorly mentioned and can be elaborated to make it easily understandable for the readers. | We have elaborated as suggested to make it more understandable |
| 3. Why the odds ratio in the case of ARDs on Chest X-ray is so high, how to explain it? Can the authors describe how they analysed the odds ratio in the result section? | Of the 399 patients in the study, poor outcomes (death or ventilation) was noted in 30.  8 of the 30 who had poor outcomes had ARDS; only 3 out of the 369 who had good outcomes had ARDS.  This considerable difference is the possible reason for the very high Odds Ratio.  We have described this in results section. |
| 4. There are several studies that suggest that one or more comorbidity condition leads to higher rate of poor outcome. However, this study found that co-morbidities is not associated with the poor outcomes. How authors will explain this? Can they provide any further supporting evidence in this regard? | We agree that several studies have identified such association with co-morbidities.  The absence of association in our case could be due to the outcomes chosen. We have included both mechanical ventilation and death as poor outcomes since we thought these are important outcomes in hospitalised patients.  Different studies have used various outcomes (such as severe disease or death alone) explaining the diversity in results.  The other explanation is the high prevalence of co-morbidities in the general population in this area. Many studies have shown that diabetes and hypertension are highly prevalent in general population in India, especially South India.  In this study, overall around 50% of patients were diabetic and 44% hypertensive. Therefore it was unable to detect any significant difference between those with and without poor outcomes. |
| 5. Table 4: Can the authors add one more column to show the %age of each variables in total population. It will be easier for the readers to read and compare the data. | This column has been added and table is now modified as table 5; however since there are few continuous variables, these could not be represented in percentages. |

**Reviewer 2**

| **Reviewer’s comments** | **Author’s responses** |
| --- | --- |
| 1. The score of 5 has been used to predict the prognosis in the patients. The authors have also stated that the AUC for a score of 5 was better than 6. How have the authors substantiated this finding ? Details may be sent | We thank the reviewer for this observation.  We have now included the comparison of diagnostic accuracy measures of the two cut-offs (5 and 6). This has been added as a table (Table 4) in the manuscript.  The sensitivity of score 5 or more was 93.3% compared to 90% for score of 6 or more. Since the score is proposed to be used as a screening tool for identifying patients who are likely to have poor outcomes, we chose the score cut off with higher sensitivity. |
| 2. Other co-morbidities have also been stated in the results and discussion section. Will the inclusion of these as stated for Fig-2 and table -5 change the score to more than 5 as a predictor of poor outcome on THE NEWS-2 scoring system | The standard NEWS2 score is validated only for the clinical variables included and does not include any of the co-morbidities like hypertension, diabetes etc. therefore we could not include them into the score.  Further, studies have shown that adding pre-existing co-morbidities to the model made no difference to risk prediction. This has been mentioned in discussion section. |
| 3. The authors have not included laboratory parameters for the NEWS-2 score. How would the NEWS-2 score compare with another scoring system that combines laboratory with clinical parameters for predicting poor outcome | The NEWS2 score is a clinical bedside scoring that includes only the respiratory rate, oxygen saturation (SpO2), need for supplemental oxygen, pulse rate, level of consciousness and temperature.  Besides the objective of our study was to determine the ability of NEWS2 score to predict poor outcomes and therefore we did not compare it with any other scores that use laboratory parameters. The advantage of NEWS2 score is that since it does not include laboratory parameters, it can be used readily in resource poor and emergency settings as well. |
| 4. The conclusion is withered and needs to be toned based on the definite results obtained in the study | Thank you for the observation. We have modified the conclusion in alignment with the results of the study. |
