# Supplementary material for: National Early Warning Score 2 (NEWS2) to predict poor outcome in hospitalised COVID-19 patients in India
Source: PLoS One. 2021 Dec 15;16(12):e0261376. doi: 10.1371/journal.pone.0261376 (PMC8673675; doi:10.1371/journal.pone.0261376)
Supplement: Attachment — Submitted filename: Response to Reviewers.docx [file pone.0261376.s004.docx]

**Reviewer 1**

| **Reviewer’s comments** | **Author’s responses** |
| --- | --- |
| 1. In the revised version still the result section is not elaborated. Thus it is difficult to understand | We regret that the previous revision failed to improve understanding.  We have added more details on number of patients who had score of 5 and above and went on to develop poor outcomes. Similar description has been given for score of 6 and above. We hope these will improve the understanding of the section.  Please see:  Lines 166 and 167  Lines 176 to 180  Lines 183 to 185 |
| 2. How the sensitivity and specificity were determined? Can the authors describe the calculations in the method section? How the sensitivity and specificity was calculated for the poor outcome? What are their significance? Can authors describe this in the result section? | Sensitivity refers to a test's ability to designate an individual with disease as positive. In this study it meant the ability of NEWS2 score to identify the proportion of people with the disease who will have a poor outcome. A highly sensitive test means that there are few false negative results, and thus fewer cases of disease are missed and is good at including most people who have the condition.  In this study, in order to calculate the sensitivity of a score of 5, we determined the proportion of patients with a baseline score of 5 and above who developed poor outcome. Poor outcome was defined as death or mechanical ventilation (mentioned in methods). Similarly, sensitivity was calculated for score of 6.  On the other hand, Specificity refers the ability of a test to correctly identify people without the disease. In this study it meant the ability of NEWS2 score to identify the proportion of people with the disease who will have a better outcome.  Hence in this study, in order to find the specificity of score of 5, we determined the proportion of patients with a baseline score of 5 and above who did not develop a poor outcome (or had good outcome). Same method was used for specificity of score 6.  We have added this explanation in the methods section and also elaborated the calculation in results and added supplementary tables (S2 tables).  Please see:  Lines 183 and 184. |
| 3. Line 160-168 needs more clarifications | In this study Table 4 and lines 160 – 166 explains about best cut off for the NEWS2 score.  We have now added descriptions of how the calculations were made.  Please see:  Lines 176 to 180  Lines 184 and 185  Supplementary tables 1 and 2 |
| 4. How the Odd ratio was calculated (line 192) is not clear. Can the authors describe it in the method section or in the result section? | The odds ratio quantifies the relationship between an exposure and a disease and tells us how much higher the odds of exposure are among cases than among controls. In this study those who need for mechanical ventilation or death within 28 days (i.e., Poor outcome) are considered as the case-patients and patients discharged (i.e., Good outcome) are considered as the control-patients. Exposure status is defined as findings of the Chest X-ray viz., ARDS and no ARDS. Logistic regression, a standard technique to calculate odds ratio, was employed to quantify the relationship between Chest X-ray and poor outcome and expressed as Odds ratio with 95% Confidence intervals and p values.  This information has been added in methods section covering Odds ratio calculation for various variables.  Please see:  Lines 122 to 130 |
| 5. Legend for both the figures should be mentioned to understand the figures. The explanation for Figure 2 is not clear. What the AUC is mentioning here? Is it for score 5 or 6? | Legend for figure 1 has been elaborated.  Lines 105 and 106  Regarding Legend for figure 2:  The Area Under the Curve (AUC) is the area under the receiver operating characteristic curve (ROC curve). It is a number between zero and one, because the ROC curve fits inside a unit square. In general, an AUC of 0.5 suggests no discrimination (i.e., ability to diagnose patients with and without the disease or condition based on the test), 0.7 to 0.8 is considered acceptable, 0.8 to 0.9 is considered excellent, and more than 0.9 is considered outstanding. Thus higher the AUC, the better the performance of the model at distinguishing between the positive and negative cases. In this study the AUC to predict poor outcome i.e., need for mechanical ventilation or death within 28 daysbased on NEWS2 score at admission was 0.887 which is considered excellent.  The sensitivity and specificity can be calculated for each cut off value of the test score and it does not depend upon AUC. In other words, AUC is not for a specific cut off score. The typo error “for this cut off” has therefore been removed since this is not applicable for AUC.  We regret the confusion caused by the previous wording.  Please see:  Line 187 and 188. |
| 6. Line 231: been a tough ask to predict: change ask to task | We thank the reviewer for identifying this mistake. We have now changed it.  Please see:  Line 273 |
| 7. As the current study did not find any association of the comorbidity with the poor outcomes, a statement should be made in the discussion section. | We thank you for this suggestion.  In response to a similar suggestion in the first review, we have already added this in the discussion.  Please see:  Line 338 to 343. |
| 8. The conclusion can be more elaborative, should present numbers. This will help the readers to understand the concluding remarks of the article | We thank you for the valuable suggestion. We have now added the numbers pertaining to sensitivity and specificity which are the primary outcomes of the study. We have not included more details as it may be a repetition of results.  Please see:  Line 354 to 356 |
